# A push-pull system to reduce house entry of malaria mosquitoes

**DOI:** 10.1186/1475-2875-13-119

**Published:** 2014-03-27

**Authors:** David J Menger, Bruno Otieno, Marjolein de Rijk, W Richard Mukabana, Joop JA van Loon, Willem Takken

**Affiliations:** 1 Laboratory of Entomology, Wageningen University, P.O. Box 8031, 6700, EH Wageningen, The Netherlands; 2 International Centre of Insect Physiology and Ecology, P.O. Box 30772, GPO Nairobi, Kenya; 3 School of Biological Sciences, University of Nairobi, P.O. Box 30197, 00100, GPO Nairobi, Kenya

**Keywords:** Mosquitoes, Malaria, Vector control, Repellent, Push-pull

## Abstract

**Background:**

Mosquitoes are the dominant vectors of pathogens that cause infectious diseases such as malaria, dengue, yellow fever and filariasis. Current vector control strategies often rely on the use of pyrethroids against which mosquitoes are increasingly developing resistance. Here, a push-pull system is presented, that operates by the simultaneous use of repellent and attractive volatile odorants.

**Method/Results:**

Experiments were carried out in a semi-field set-up: a traditional house which was constructed inside a screenhouse. The release of different repellent compounds, para-menthane-3,8-diol (PMD), catnip oil e.o. and delta-undecalactone, from the four corners of the house resulted in significant reductions of 45% to 81.5% in house entry of host-seeking malaria mosquitoes. The highest reductions in house entry (up to 95.5%), were achieved by simultaneously repelling mosquitoes from the house (push) and removing them from the experimental set-up using attractant-baited traps (pull).

**Conclusions:**

The outcome of this study suggests that a push-pull system based on attractive and repellent volatiles may successfully be employed to target mosquito vectors of human disease. Reductions in house entry of malaria vectors, of the magnitude that was achieved in these experiments, would likely affect malaria transmission. The repellents used are non-toxic and can be used safely in a human environment. Delta-undecalactone is a novel repellent that showed higher effectiveness than the established repellent PMD. These results encourage further development of the system for practical implementation in the field.

## Background

Mosquitoes are the dominant vectors of pathogens that cause infectious diseases such as malaria, dengue, yellow fever and filariasis [1,2]. Vector control strategies are aimed at disrupting transmission cycles and are an important tool in the prevention of these diseases. Current vector control strategies often rely on the use of insecticide-treated nets (ITNs) and indoor residual spraying (IRS) [3,4]. However, the rapidly increasing resistance of mosquitoes to the active chemicals on which these strategies depend implies a serious limitation of their efficacy [5-8].

The literature provides examples of various alternative vector control tools that could be employed as supplements to, or possibly even as replacements of, ITNs and IRS (reviewed by [9]). A tool which has previously proven its value in the context of agricultural pest management is the so called ‘push-pull system’ [10]. A push-pull system manipulates the behaviour and/or distribution of pest insects by the simultaneous use of repellent and attractive stimuli. In this paper, a push-pull system is introduced, that is directed at the major African malaria vector *Anopheles gambiae sensu stricto (s.s.)*. The system is based on removal trapping and the release of spatial repellents.

Removal trapping is a strategy that aims at reducing the target insect population with attractive traps placed in strategic locations. This strategy is effective against tsetse flies (*Glossina* spp.), which transmit trypanosomiasis (sleeping sickness), and against other disease vectors [11]. Recent laboratory and field experiments have led to the development of odour blends based on ammonia, L-lactic acid and carboxylic acids which, in combination with carbon dioxide (CO_2_), can be used as baits to effectively trap tropical mosquitoes, including malaria vectors [12-18].

Repellents can be applied topically for personal protection, e.g. the widely used insect repellent DEET (N,N-diethyl-meta-toluamide), but can also be dispersed spatially to protect a space, e.g. the burning of repellent-impregnated coils, candles that contain certain essential oils or leaves of specific tree species [19-22]. Repellents that exhibit a spatial effect may be considered for inclusion in a push-pull system.

The use of push-pull tactics fits within the emerging view that vector control strategies should be expanded beyond insecticide-dependent methods [4]. Combining the mechanisms of attraction and repellency has the potential to result in a synergistic effect [10]. By ‘pushing’ mosquitoes away from certain places using repellents, one could stimulate their movement towards other places where they are ‘pulled’ into traps baited with attractive cues. Now that highly attractive synthetic odour blends that mimic human scent are at the disposal of the scientific community, the remaining challenge lies in the development or selection of effective spatial repellents directed at the target group.

In this paper, two experiments are presented in which it is demonstrated how (1) a push-pull system was employed in a semi-field situation where it successfully reduced house entry of the predominant malaria vector in sub-Saharan Africa, *An. gambiae s.s.* and (2) this push-pull system was improved with the introduction of a novel mosquito repellent that displays a superior spatial effect.

## Methods

### Mosquitoes

The mosquitoes (*An. gambiae s.s.*, Mbita strain; henceforth termed *An. gambiae*) were reared under ambient atmospheric conditions in screenhouses (larvae) and indoors (adults) at the Thomas Odhiambo Campus (TOC) of the International Centre of Insect Physiology and Ecology (*icipe*) located near Mbita Point township in western Kenya. Mosquito eggs were placed in plastic trays containing filtered water from Lake Victoria. All larval instars were fed on Tetramin® baby fish food which was supplied thrice per day. Pupae were collected daily and placed in mesh-covered cages (30 × 30 × 30 cm) prior to adult emergence. Adult mosquitoes were fed on 6% glucose solution through wicks made from adsorbent tissue paper.

Female mosquitoes of 3 – 6 days old since eclosion that had no prior access to blood were used for the semi-field experiments. The mosquitoes were collected from the colony at 12:00 h each day and stored for 8 h in the colony room with access to water on cotton wool. Within 15 min before the start of the experiment the cups with the mosquitoes were transported to the experimental set-up.

### Description of the set-up

**Figure 1:**
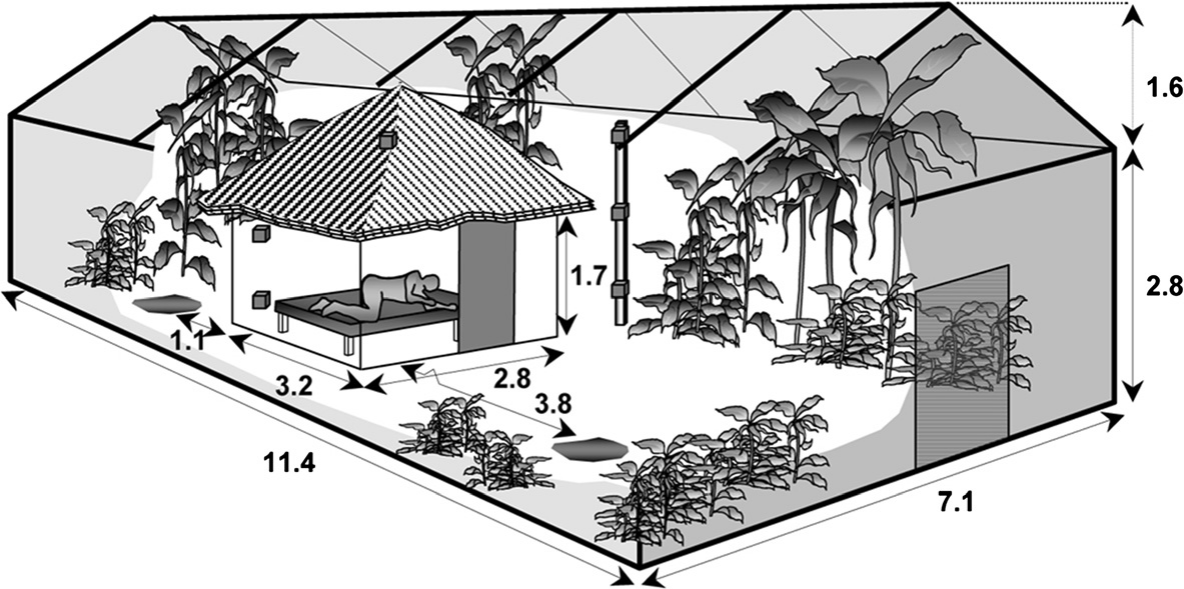
**The MalariaSphere; a screenhouse with a traditional house constructed inside (image copied from **[23]**)**

The experiments were conducted at the Mbita Point Research & Training Centre of *icipe* in Kenya. Experiments took place in the MalariaSphere (Figure 1), a screenhouse into which a traditional house was built surrounded by natural vegetation [23]. The traditional house possesses an eave, through which mosquitoes that are released into the screenhouse may enter, as they would do in a natural situation when an attractive host is present inside [24]. The MalariaSphere was set up as described [23], with the only modification that no breeding sites were present.

### Experimental design

Both experiments explored the effects of attractant-baited traps and the dispersal of repellents around the traditional house. Four different set-ups were tested during experiment 1 and eight different set-ups were tested during experiment 2. During all tests, one attractant-baited trap (see below) was placed inside the experimental house to represent a human being. The house entry of the mosquitoes was measured by the number of mosquitoes caught by the trap inside the house.

Each night at 20:00 h, 200 female mosquitoes were released into the MalariaSphere. At 6:30 h the next morning the experiment was terminated by closing and switching off the ventilators of all traps. The traps were then placed in a freezer for several minutes to inactivate the mosquitoes, after which the numbers of trapped mosquitoes were determined.

#### Experiment 1

**Figure 2:**
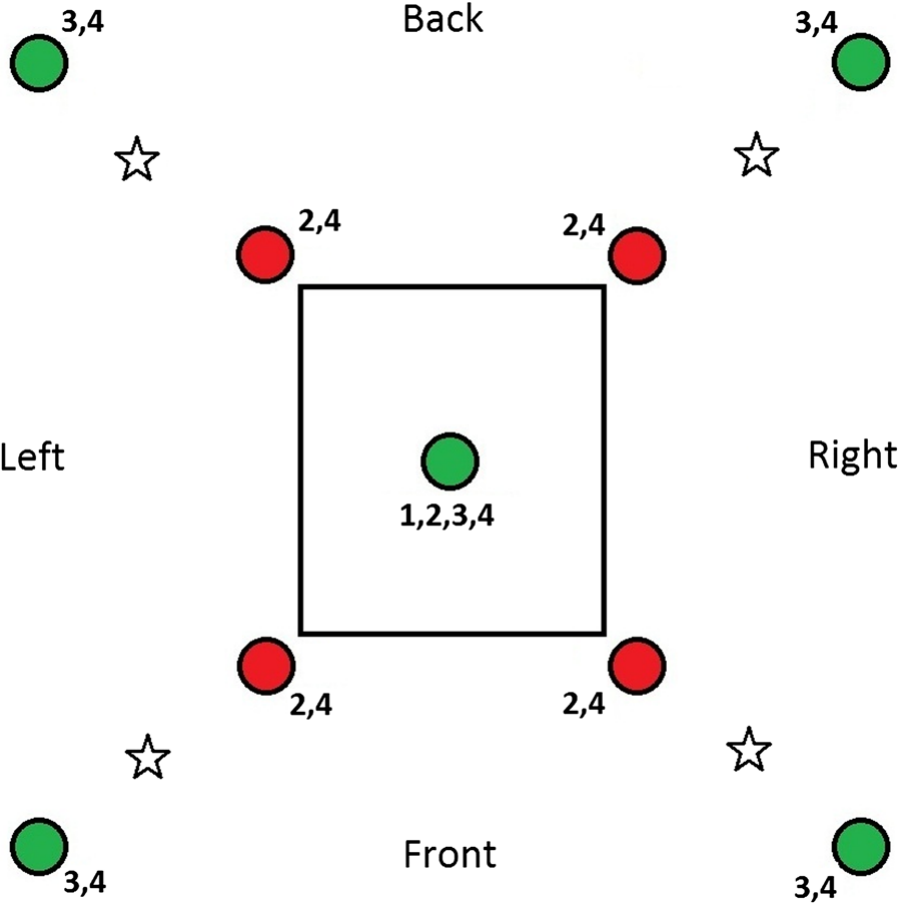
**Experimental set-up of experiment 1.** Green represents an MMX trap baited with attractant, red represents an MMX trap dispersing the repellent. Asterisks indicate the mosquito release points. Numbers indicate the treatments at which the trap or dispenser was present (see also Table 1).

The four set-ups that were tested during experiment 1 included: (1) a control set-up in which only the attractive trap inside the house was present, (2) a push-only situation in which a repellent was released from the four corners of the house, (3) a pull-only situation in which four attractant baited-traps were positioned around the house and (4) a situation in which the total push-pull system was set up with both the repellent and the attractant components in place. See Table 1 for the presence/absence of the specific traps during the treatments and Figure 2 for an overview of their positions. Each set-up was tested during eight different nights, thus a total of 32 tests was carried out during the same number of nights. The order of the tests was not fully randomized in order to minimize the risk of contamination of the MalariaSphere with the used odours. The repellent compound selected for this experiment was para-menthane-3,8-diol (PMD) [25]. Nylon strips were impregnated with a 40% solution of commercially available Citriodiol^TM^ (containing > 64% PMD) as described below. At the start of each test, the mosquitoes were released from four different spots around the house (50 mosquitoes per spot), see Figure 2.

**Table 1:** Placement of attractants and repellents in experiment 1 (Yes/No)

**Treatment**	**Attractant inside**	**Attractant outside**	**Repellent outside**
1	Y	N	N
2	Y	N	Y
3	Y	Y	N
4	Y	Y	Y

See also Figure 2.

#### Experiment 2

**Figure 3:**
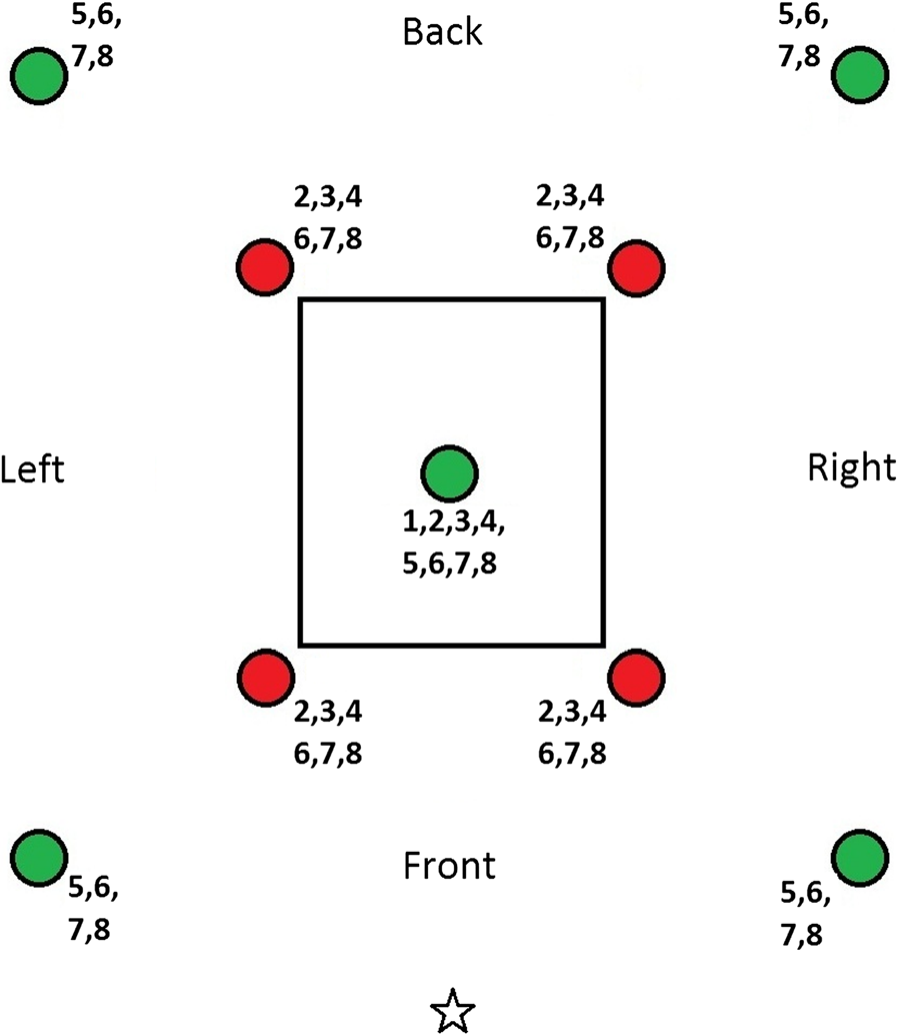
**Experimental set-up of experiment 2.** Green represents an MMX trap baited with attractant, red represents an MMX trap dispersing the repellent. The asterisk indicates the mosquito release point. Numbers indicate the treatments at which the trap or dispenser was present (see also Table 2).

During experiment 2, eight different set-ups were tested. This study compared the effect of three different repellents in push-only situations as well as in situations in which both a repellent and the attractive blend were released; see Table 2 and Figure 3 for a comprehensive overview of which repellent compound was used during the different tests, the presence/absence of the repellent and attractive components and their positions. Each set-up was tested during six different nights, thus a total of 48 tests was carried out, during the same number of nights. The order of the tests was not fully randomized in order to minimize the risk of contamination of the MalariaSphere with the used odours. PMD (see Experiment 1), catnip essential oil (e.o.) [26,27] and delta-undecalactone (dUDL; patent pending) [28] were used as repellents. Strips were impregnated with 40% solutions (catnip e.o. and dUDL were dissolved in paraffin oil) as described below. During Experiment 2, all 200 mosquitoes were released from one central point between the entrance of the screenhouse and the experimental hut (see Figure 3).

**Table 2:** Placement of attractants and repellents in experiment 2 (Yes/No)

**Treatment**	**Attractant inside**	**Attractant outside**	**Repellent outside**
1	Y	N	N
2	Y	N	Y (PMD)
3	Y	N	Y (Catnip)
4	Y	N	Y (dUDL)
5	Y	Y	N
6	Y	Y	Y (PMD)
7	Y	Y	Y (Catnip)
8	Y	Y	Y (dUDL)

See also Figure 3.

### Attractant-baited traps

Mosquito Magnet® X (MM-X) traps [29,30] were baited with CO_2_ and a five-compound odour blend, which simulates the smell of a human foot [18,28]. The individual compounds of the attractive blend were released from nylon strips (cut from panty hoses: 90% polyamide, 10% spandex, Marie Claire®) [31]. Concentrations were optimised for this set-up and release method: ammonia (2.5% in water), L-(+)-lactic-acid (85%), tetradecanoic acid (0.00025 g/l in ethanol), 3-methyl-1-butanol (0.000001% in water) and butan-1-amine (0.001% in paraffin oil) (see Table 3). Nylon strips (26.5 cm × 1 cm) were impregnated with the attractive compounds by dipping three strips in 3.0 ml of compound in a 4 ml screw top vial (Experiment 1) or by dipping individual strips into an Eppendorf tube containing 1 ml of solution (Experiment 2). Before use, strips were dried for 9–10 h at room temperature. During experiment 1 for every experimental night a set of freshly impregnated strips was used. During experiment 2 strips were used for a maximum of 12 consecutive nights. During daytime, the strips were packed in aluminium foil and stored at 4°C in a refrigerator.

**Table 3:** Composition of the attractive blend

**Compound**	**Concentration**	**Solvent**
Ammonia	2.5% (v/v)	Water
L-(+)-lactic acid	85% (w/w)	Water
Tetradecanoic acid	0.00025 g/l	Ethanol
3-Methyl-1-butanol	0.000001% (v/v)	Water
Butan-1-amine	0.001% (v/v)	Paraffin oil

The five strips were held together with a safety pin and hung in the outflow opening of the MM-X trap using a plastic covered clip. CO_2_ was produced by mixing 17.5 g yeast with 250 g sugar and 2.5 L water [32] and released from the MM-X trap together with the odours. MM-X traps equipped with the attractive blend were positioned with the outflow opening at the optimal height of 15–20 cm above the floor surface [33].

### Dispersal of the repellents

To disperse the repellents, MM-X traps were used of which the suction mechanism was disabled; leaving only the outflow mechanism functional [15]. The repellent compounds were applied to nylon strips identically to the attractants. However, because of their volatility the strips with repellent were dried for only 1 h (Experiment 1) or 10 min (Experiment 2). One repellent strip was used per MM-X trap. Freshly prepared strips were used each night. The MM-X traps that dispersed the repellent were hung from the lowest part of the roof of the traditional house, with the outflow opening about 1 m above the floor, to intercept mosquitoes that would enter through the eaves of the experimental hut.

### Statistical analysis

For both experiments, the trap catches inside and (when applicable) outside the experimental house were compared between all treatments. The Shapiro-Wilk test was used to test the normality of the data and Levene’s test was used to test for equality of variances. Subsequently, the differences between trap catches inside the house in Experiment 1 were analysed using analysis of variance (ANOVA) followed by Bonferroni post-hoc tests. Trap catches outside were compared using an independent-samples t-test. Differences between trap catches inside the house in Experiment 2 were analysed using ANOVA followed by Games-Howell post-hoc tests. Trap catches outside the house were compared using ANOVA followed by Bonferroni post-hoc tests.

## Results

### Experiment 1

**Figure 4:**
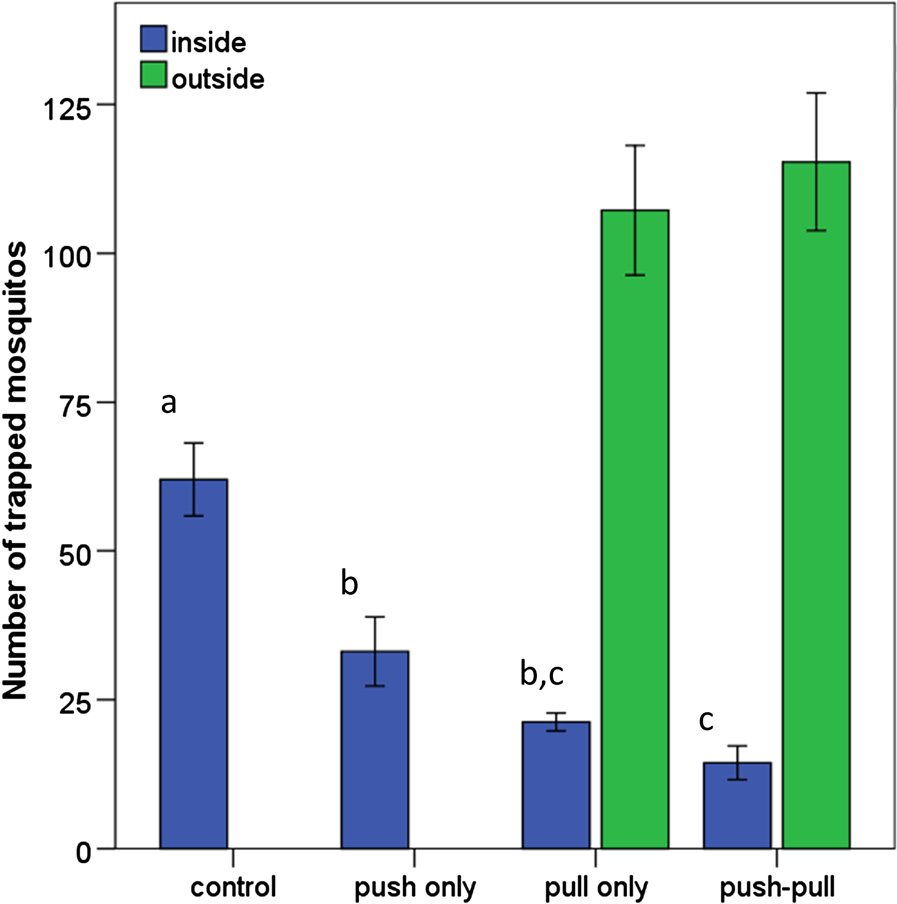
**Mean number of mosquitoes trapped inside and, when applicable, outside the experimental house.** For all treatments n = 8, error bars indicate the standard error of the mean. Bars not sharing the same character are significantly different at α = 0.05 with Bonferroni post-hoc tests.

During the control tests, the attractant-baited trap inside the house caught on average 62.0 (SEM 8.7) or 31.0% of the released mosquitoes. The release of PMD (push only), removal trapping (pull only) and the combination of both strategies (push-pull) all significantly reduced the house entry of *An. gambiae* compared to the control situation (ANOVA: F = 21.53, df = 3, p < 0.001; Bonferroni post-hoc tests at α = 0.05, see Figure 4).

When PMD was released from the four corners of the house, the number of trapped mosquitoes dropped to 31.1 (8.2); a reduction of nearly 50%. With four attractant-baited traps placed around the house, even fewer mosquitoes entered the house, with the trap indoors catching only 21.3 (2.1) mosquitoes on average. The four traps outdoors caught 107.3 (15.4) mosquitoes or 53.7% of the total number released. With both the push and the pull components in place, the number of mosquitoes trapped indoors was lowest, with only 14.4 (4.0) mosquitoes on average, or 7.2% of the total number released. This implies a reduction of more than 75% compared to the control treatment. The traps outdoors caught an average of 115.4 (16.3) mosquitoes in the push-pull scenario.

### Experiment 2

**Figure 5:**
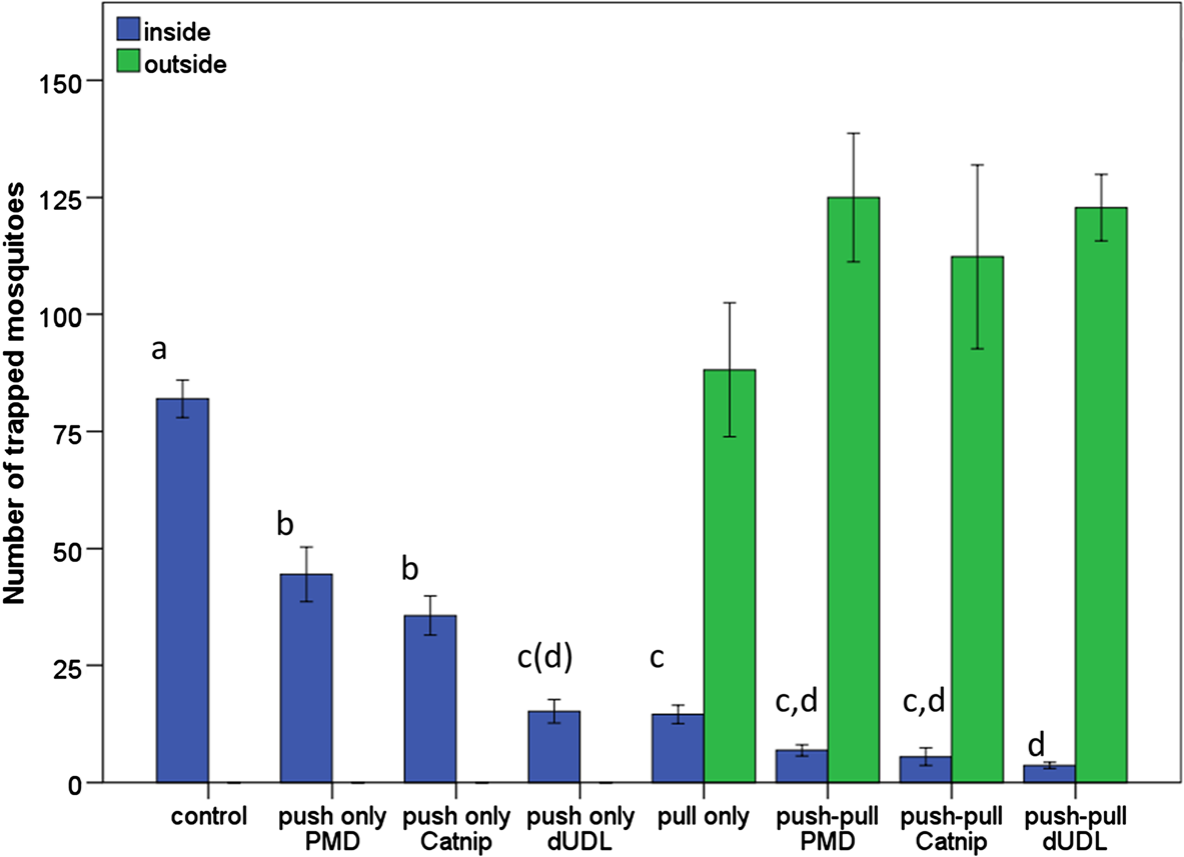
**Mean number of mosquitoes trapped inside and, when applicable, outside the experimental house.** For all treatments n = 6, error bars indicate the standard error of the mean. Bars not sharing the same character are significantly different at α = 0.05 with Games-Howell post-hoc tests. (d): p = 0.05081 for the comparison between the push-only dUDL treatment and the push-pull dUDL treatment.

In the absence of repellent dispensers or removal trapping, the attractant-baited trap inside the house caught 82.0 (4.0) mosquitoes on average; 41.0% of the total number released. As in the previous experiment, all treatments significantly reduced the number of mosquitoes trapped in the experimental house (ANOVA: F = 70.08, df = 7, p < 0.001; Games-Howell post-hoc tests at α = 0.05, see Figure 5).

The push-only treatment in which delta-undecalactone was dispensed caused a significantly stronger reduction (81.5%) than the treatments with PMD or catnip e.o (45.7% and 56.5% resp.), of which catnip e.o. performed slightly (ns) better. Removal trapping (pull only) led to a 82.3% reduction, with the trap inside the house catching only 14.5 (2.0) mosquitoes on average. The push-pull treatment employing delta-undecalactone as a repellent provided the strongest reduction, 95.5%; only 3.7 (0.7) mosquitoes were caught inside the house on average; 1.9% of the total number released. The total number of mosquitoes trapped outdoors did not differ significantly between the treatments that included removal trapping.

## Discussion

### Efficacy of the push-pull system

An attractant-baited trap placed inside a traditional house caught 31% (experiment 1) to 41% (experiment 2) of the mosquitoes released in the screenhouse. Therefore, host-seeking female mosquitoes must have entered the house attracted by the combination of odour + CO_2_ that was deployed to mimic a potential host. This confirms that the odour blend + CO_2_ functions analogous to a human host in terms of inducing house entry as a component of host-seeking behaviour [15,18].

The release of PMD from the four corners of the house resulted in a significant reduction of over 45% in house entry of host-seeking mosquitoes. Therefore, anyone being indoors would have received fewer mosquito bites under this treatment. Experiment 2 showed that this effect improved significantly (to 81.5%) when PMD was replaced by delta-undecalactone.

The placement of attractant-baited traps around the house significantly reduced the number of mosquitoes trapped inside the house, in both experiments. Instead of entering the house, a high percentage (53.7% and 44.1% resp.) was lured into the traps placed outdoors.

These examples show that it is feasible to trap or repel host-seeking mosquitoes before house entry, thereby rendering protection to the house occupants. The highest reductions in house entry (up to 95.5%), and thus the highest degrees of protection, were achieved by simultaneously repelling mosquitoes from the house (push) and removing them from the experimental set-up by trapping (pull). Although outdoor trap catches were slightly elevated when both push and pull were present, compared to pull only, there was no statistical indication that a greater push led to a greater pull or vice versa. Rather than a synergistic interaction between both components, the attractant and repellent seem to have independent effects that, by their different modes of action, complement each other.

### Spatial repellency

The results also show that PMD, catnip e.o. and delta-undecalactone, had an effect on the mosquitoes over a large distance, as the places from where the repellents were dispensed were approx. 3 m apart. Released in an appropriate way, in the present experiments by active dispersion from nylon fabric, these compounds thus act as spatial repellents.

PMD has previously been shown to be an effective repellent against mosquitoes of several genera, including vectors of human disease ([25] and references therein). Catnip e.o. has also been reported as an insect repellent, with proven effect on mosquito species of several genera including *Aedes, Anopheles* and *Culex*[26,27,34,35].

Delta-undecalactone was first identified in studies of the olfactory receptors of *An. gambiae* using *ex vivo* heterologous olfactory receptor expression assays [36] and *in vivo* electrophysiological studies on antennal sensilla [37-39]. Subsequently, it was selected for tests in a repellent bioassay, where it showed an equal or higher level of repellency than DEET [28]. The superior spatial repellent effect it displayed in this experiment underlines its potential as a new repellent that may be used for the control of mosquito vectors of disease. Because delta-undecalactone is a natural product present in edible fruits and dairy products [40,41], regulatory issues concerning its use as a repellent are expected to be limited making it a suitable compound for inclusion in vector-control programmes.

### Field implementation

The outcome of this study suggests that a push-pull system based on odorant volatiles may successfully be employed to target mosquito vectors of human disease. Reductions in house-entry of the magnitude observed in this study, would likely affect malaria transmission, especially in areas where mosquito densities are low and malaria risk is directly related to the entomological inoculation risk [42]. So far, house entry reductions of this magnitude are only known for pyrethroid insecticides (e.g. [43,44]). The results presented here justify the decision to keep working on a field-proof push-pull system based on a combination of non-pyrethroid repellents and attractants.

The usefulness of push-pull systems for control of mosquito-borne diseases will not only depend on their efficacy in repelling and trapping mosquitoes, but also on their applicability and cost-effectiveness [45]. For malaria control, vector control measures should be affordable and usable in rural African settings. In its current shape, employing up to nine electrically-powered MM-X traps, the push-pull system presented here does not meet these requirements. Therefore, follow-up experiments are planned to further optimize this system and explore the practical implementation of an odour-based push-pull system that is less dependent on electric power.

Attractant odour baits have been reported that can be formulated to last for several months [46]. Odour-baited traps can be operated and maintained by house owners, preferably through a community approach, improving the sustainability of this vector control method. Studies on repellent formulation and passive distribution mechanisms are still required.

Finally, this system may also be considered in areas where most of malaria transmission occurs outdoors [47,48], where it is expected to increase the efficacy of existing methods such as ITNs and IRS that do not target host-seeking mosquitoes outside the house.

## Conclusion

This study shows a strong spatial effect of PMD, catnip oil and delta-undecalactone, when dispensed around a house in a semi-field set-up. Combined with an attractant in a push-pull strategy, the volatile repellents caused highly significant reductions in house entry of the major African malaria vector *An. gambiae.* These results encourage further development of the system for practical implementation in the field.

## Competing interests

The authors declare that they have no competing interests.

## Authors’ contributions

DM, MdR, RM, JvL and WT designed the experiments. DM, BO and MdR conducted the experiments. DM analysed the data. DM, JvL and WT wrote the manuscript. All authors read and approved the final manuscript.
